# DEVELOPING A STRATEGY TO SCREEN FOR CAREGIVER NEGLECT: EXAMINING EXISTING PUBLISHED TOOLS

**DOI:** 10.1093/geroni/igae098.2055

**Published:** 2024-12-31

**Authors:** E-Shien Chang, Alyssa Elman, Daniel Baek, Tony Rosen

**Affiliations:** Weill Cornell Medical College, New York, New York, United States; Weill Cornell Medicine, New York, New York, United States; Weill Cornell Medicine, New York, New York, United States; Weill Cornell Medicine, New York, New York, United States

## Abstract

Screening for elder mistreatment among older adults with dementia may be helpful, but the value of existing tools is not supported by evidence. Shifting the focus to individual sub-types of mistreatment may provide improved approaches to screening and ultimately to intervention. We have chosen to focus on caregiver neglect, the subtype associated with highest mortality and particularly common in dementia. As a preliminary step to develop a novel strategy to screen for caregiver neglect, we identified all existing published elder mistreatment screening tools to find items assessing for the presence of or risk for caregiver neglect. We identified 31 screening tools, which were all reviewed by 3 team members with complementary expertise (gerontology, social work, emergency medicine). Overall, none of the tools screened only for caregiver neglect. Among them, 22 (71%) included neglect-related items consisting of patient questions, caregiver questions, and provider observations. The total percent of items within each tool assessing for neglect ranged from 18-96%. After removing identical or very similar items, a final of list 202 was created, the majority of which (67.8%) were provider observations. These items were further grouped into 42 discrete categories: 11 categories of patient questions, 10 of caregiver questions, and 21 of provider observations. These serve as a clear starting point for development of caregiver neglect screening.

